# Rehabilitation of An Analgesic Bracelet Based on Wrist-Ankle Acupuncture in Patients with Rotator Cuff Injury: A Randomized Trial

**DOI:** 10.1155/2021/9946548

**Published:** 2021-07-13

**Authors:** Wenjuan Song, Xin Wang, Jishen Zhou, Ping Shi, Wei Gu, Fanfu Fang

**Affiliations:** ^1^Department of Rehabilitation, Changhai Hospital, Naval Medical University, Shanghai, China; ^2^Department of Traditional Chinese Medicine, Naval Medical University, Shanghai, China; ^3^Institute of Rehabilitation Engineering and Technology University of Shanghai for Science and Technology, Shanghai, China

## Abstract

**Objective:**

To evaluate the effect of transcutaneous electrical nerve stimulation (C-TENS) in the rehabilitation of rotator cuff injury.

**Methods:**

A total of 60 patients diagnosed with mild to moderate rotator cuff injury were randomly divided into the control group and test group. Both groups received conventional rehabilitation treatment including physical therapy, joint mobilization, interventional therapy, and family recovery training. The test group additionally received TENS treatment on the upper area 4 or 5 of the wrist, based on the wrist-ankle acupuncture (WAA) theory. The results of the visual analogue scale (VAS), shoulder range of motion (ROM), and Constant-Murley score (CMs) were collected before and after the 1^st^ treatment and after the 5^th^ treatment. The differences of those indicators between the two groups were analyzed statistically.

**Results:**

The VAS scores of measurement points after treatment were all improved compared with those at baseline. There was a significant difference between the two groups after the 1^st^ and the 5^th^ treatment (*p* < 0.05), and the improvements in the test group were better than those in the control group. The ROM of flexion, extension, abduction, adduction, internal rotation, and external rotation after the 1^st^ treatment and 5^th^ treatment in both groups were all improved compared with those at baseline. There was no significant difference between two groups. The CMs of the two groups after the 5^th^ treatment were all improved compared with those at baseline. There was no significant difference between two groups. No adverse events occurred during the treatment.

**Conclusion:**

Electrical stimulation on the wrist combined with conventional rehabilitation is more effective in relieving pain than the conventional rehabilitation alone. Electrical stimulation on the wrist combined with conventional rehabilitation has no obvious effect in improving shoulder joint mobility and shoulder function.

## 1. Introduction

Shoulder pain is a common clinical symptom that leads to limited shoulder joint movement and affects the quality of life. It is estimated that the incidence of shoulder pain among adults aged 45–64 is 2.4% [1]. Common causes of shoulder pain are adhesive arthritis and acromioclavicular joint disease [2], and about 70% of should pain cases are caused by rotator cuff injury [3]. At present, rehabilitation physiotherapy is often adopted in the treatment of mild to moderate rotator cuff injuries, by means of electrical stimulation, manual therapy, functional exercise, local pain closure, and acupuncture, to reduce pain and inflammation. As a noninvasive physical therapy, transcutaneous electrical nerve stimulation (TENS) is widely used in symptomatic treatment of pain, including hip postoperative pain [4], hemiplegic shoulder pain [5], childbirth pain [6], and cancer pain [7]. With different frequencies and intensities, the electrical stimulation can be divided into electrical stimulation on the sensory level and electrical stimulation on the motor level. Conventional TENS (C-TENS) is defined as sensory level stimulation, which can inhibit the nociceptor induced dorsal horn response by stimulating large-diameter primary afferent fibers so as to produce the analgesic effect [8]. Wrist-ankle acupuncture (WAA) is a special acupuncture therapy of traditional Chinese medicine (TCM), which follows the longitudinal axis of the limbs and just performs superficial subcutaneous needle punctures at specific points on the wrist and ankle to treat diseases. This study was guided by the theory of WAA and selected the appropriate points for low-frequency electrical stimulation to explore the rehabilitation effect of local TENS on the pain and shoulder function of the patients with rotator cuff injury.

## 2. Materials and Methods

### 2.1. Participants

A total of 60 patients with rotator cuff injury treated at the Department of Rehabilitation Medicine of Changhai Hospital between June 2017 and December 2017 were selected. Diagnostic criteria were as follows: clinical symptoms such as shoulder pain and decreased range of motion; physical examination such as tenderness in the space between the anterior shoulder and the greater tuberosity and positive result in the painful arc test, impact test, Jobe test, Lag test, or lift-off test; and MRI: according to the Zlatkin classification, imaging findings were of grades 1–3, and conservative treatment or postoperative rehabilitation can be performed after medical evaluation. Inclusion criteria were as follows: meet the diagnostic criteria for rotator cuff injury, aged 20–80 years old; no analgesic drugs, local closure, or any other treatments were used in the last 24 hours; no other chronic pain diseases; good mental state can cooperate with experimental research; and voluntarily receive treatment. Exclusion criteria were as follows: the history of tumor or tuberculosis; diseases of the heart, liver, spleen, lung, or kidney or other major viscera diseases; epilepsy, pregnancy, or infectious diseases; with pacemaker; and vascular expansion or scars or wounds on the wrist. Termination criteria were as follows: adverse reactions, including subcutaneous bleeding, electric burns, and allergies; symptoms worsen; and demanding further treatment or painkillers.

This study was a monocentric, prospective, randomized, single-blind, controlled trial. The subjects who met the inclusion criteria were assigned to the test group (receiving conventional rehabilitation training combined with local TENS) or the control group (receiving conventional rehabilitation training alone) by using a simple computerized random number generator. All the subjects were blind to the group assigning. This study was approved by the China Clinical Trial Ethics Review Board (No. ChiECRCT-2017051) and was listed on the China Clinical Trial Register (No. ChiCTR-IIR-17012275).

### 2.2. Intervention

#### 2.2.1. Test Group

Conventional rehabilitation training: ① physical therapy and joint mobilization can relieve pain through mechanical action and nerve action, maintain tissue stretchability, improve joint range of motion, and increase proprioceptive feedback. Professional rehabilitation therapists performed active and passive joint movement and joint drafting and extrusion [9]. The axial movement of the shoulder joint was carried out without pain or slight pain. Course of treatment: 2-3 times a week, 5 times in total, 2–3 weeks. ② Interferential therapy can promote blood circulation and relieve pain. Place two pairs of electrodes oppositely around the pain point, select voltage of 10–20 V, current of 10–20 mA, frequency of 75–100 Hz, suction pressure of 5–25 kPa, and treatment time of 20 minutes. Course of treatment: 2-3 times a week, 5 times in total, 2–3 weeks. ③ Family recovery training can promote the recovery of the shoulder function. Pendulum, crossover arm stretch, passive internal rotation, passive external rotation, sleeper stretch, and scapula setting can be performed with reference to the Rotator Cuff and Shoulder Conditioning Program by American Academy of Orthopaedic Surgeons. The course of training was 2–4 times a day [10].

**Figure 1:**
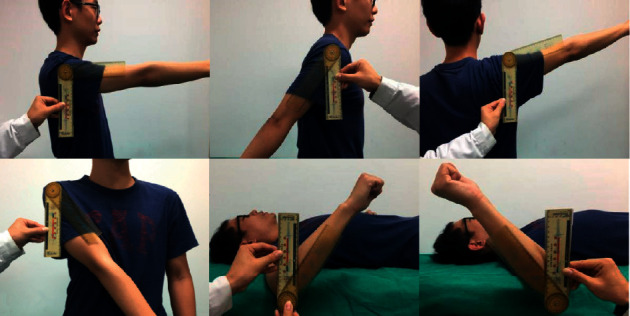
The therapist measures the patient's shoulder joint range of motion.

Local TENS treatment: ① Location: according to WAA theory, select the treatment area on the wrist corresponding to the location of shoulder pain. If the pain is at the anterior and posterior junction of the body, the electrode should be placed in the upper area 4, which is between the inner and outer edges of the radius on the thumb side. If the pain is on both sides of the body, the electrodes shall be placed in the upper area 5, which is at the center of the wrist between the radius and the ulna. ② Equipment: a wearable electrical stimulation device jointly developed by Changhai Hospital and University of Shanghai for Science and Technology (China National Invention Patent: No. 201610928117.5). ③ Operation methods: participants should wear the device during rehabilitation training. Two electrical stimulation electrodes were placed on the upper area 4 or 5 with the skin of the treatment area exposed, and the upper and lower juxtaposition method was also adopted. Therapists should select appropriate pulse parameters for the subjects, with symmetrical two-way wave, voltage of 40 V, frequency of 70 Hz, pulse duration of 200 *μ*s, current generally less than 3 mA, and treatment time of 30 minutes. Course of treatment: 2-3 times a week, 2-3 weeks (Figure 1).

#### 2.2.2. Control Group

Therapists only performed conventional rehabilitation training on the patients.

### 2.3. Outcome Measures

The time points for outcome collection are before and after the 1^st^ treatment and after the 5^th^ treatment.

#### 2.3.1. Visual Analogue Scale

Visual analogue scale (VAS) was used to evaluate the degree of rest pain. Generally, 0–10 represents various degrees of pain, “0” represents no pain and “10” represents the most severe pain.

#### 2.3.2. Shoulder Range of Motion (ROM)

Therapist measured the active movement of shoulder joint within painless range in six directions, i.e., flexion, extension, abduction, adduction, external rotation, and internal rotation, with a protractor. In the standing position, the long axis of the humerus is used as the moving arm, and the longitudinal axis of the trunk is the fixed arm; then, the flexion, extension, abduction, and adduction were measured with the acromion as the center. In the supine position, the long axis of the ulna was used as the moving arm, and the fixed arm was perpendicular to the torso plane; then, the internal rotation and external rotation were measured with the olecranon as the center.

#### 2.3.3. Constant-Murley Score (CMs) [11]

The Constant-Murley score consists of pain (15 points), activities of daily living (20 points), movement (40 points), and strength (25 points). The total score was 100 points. Pain and activities of daily living scores were given by patients themselves; movement and strength required physical assessment.

All interventions were performed under the supervision of therapists to prevent the complications of the rehabilitation training or TENS stimulation. The subjects were asked to inform the therapists if they felt any discomfort after TENS.

### 2.4. Statistical Analysis

SAS 9.4 was used for statistical analysis. Descriptive statistics, e.g., mean and standard deviation (SD), were adopted to analyze participants' demographic data. GEE was used to evaluate VAS, ROM, CMs, and other trends over time. Measurement data in the results were expressed by median, the first quartile (*Q*_1_), and the third quartile (*Q*_3_) and were evaluated by the Wilcoxon ranks test, while the enumeration data were processed by the chi-square test. The *p* value less than 0.05 considered as the difference was statistically significant.

## 3. Results

### 3.1. Comparison of Demographic Data of the Subjects in the Two Groups

There was no statistically significant difference between the two groups in terms of sex, age, MRI grade, surgical status, and clinical baseline data (Table 1).

### 3.2. Comparison of VAS scores

The difference in the trend of VAS scores over time between the two groups of patients was statistically significant (Wald*χ*^2^ = 180.82, *p* < 0.000). The VAS scores after the 1^st^ treatment and the 5^th^ treatment in both groups were all improved compared with those at baseline. There was a significant difference between the two groups after the 1^st^ and the 5^th^ treatment (*p* < 0.05), and the scores of the test group were better than those of the control group (Table 2).

### 3.3. Comparison of ROM

The difference in the trend of ROM changes over time between the two groups was statistically significant (flexion: Wald*χ*^2^ = 235.61, *p* < 0.000; extension: Wald*χ*^2^ = 78.19, *p* < 0.000; abduction: Wald*χ*^2^ = 233.02, *p* < 0.000; adduction: Wald*χ*^2^ = 88.36, *p* < 0.000; internal rotation: Wald*χ*^2^ = 169.36, *p* < 0.000; and external rotation: Wald*χ*^2^ = 114.64, *p* < 0.000). The ROM of flexion, extension, abduction, adduction, internal rotation, and external rotation after the 1^st^ and the 5^th^ treatment in both groups were all improved compared with those at baseline. There was no significant difference between two groups (*p* > 0.05) (Table 3).

### 3.4. Comparison of CMs

The difference in the trend of CMs over time between the two groups was statistically significant (pain: Wald*χ*^2^ = 56.59, *p* < 0.000; ADL: Wald*χ*^2^ = 90.00, *p* < 0.000; ROM: Wald*χ*^2^ = 126.76, *p* < 0.000; strength: Wald*χ*^2^ = 21.82, *p* < 0.000; and the total score: Wald*χ*^2^ = 221.11, *p* < 0.000). The CMs subscale scores of the two groups after the 5^th^ treatment were all improved compared with those at baseline, and there was no significant difference between two groups (*p* > 0.05) (Table 4).

### 3.5. Adverse Event

There are no skin allergies, scalds, burns, tingling, pain, muscle numbness, other physical damage, leakage, output disfunction, unstable output, electrode piece/lead wire damage, or other equipment malfunction during the treatment.

## 4. Discussion

Rotator cuff injury is the degeneration and tear of the rotator cuff muscle at the stop point of the humerus. It is a common sports injury and a common degenerative injury for manual workers and middle-aged and elderly people. Its incidence increases with age [12]. Its clinical manifestations are neck and shoulder pain, shoulder weakness, and limited shoulder joint movement. Rotator cuff injury is the result of the combined effect of internal and external factors. The internal factors include insufficient blood supply to the rotator cuff tendon and the special position and function of the supraspinatus tendon; the external factors include the repeated use of the shoulder joint, subacromial impact, and the severity of shoulder injury [13]. The free nerve fibers, which are rich in the subacromial tissues (subacromial capsule, rotator cuff tendon, and biceps long head tendon), are activated in the case of inflammation or injury and thus led to shoulder pain [14]. In addition to nociceptive pain, studies have shown that 10.9% of patients with rotator cuff injury may also suffer from neuropathic pain [15]. Peripheral nerves, such as suprascapular nerve, can be injured by inflammation or stimulated by prostaglandin, substance P, and other chemicals released from damaged cells and inflammatory cells, leading to the sensitization of nociceptors which amplified its afferent nerve signals, and then resulted in peripheral sensitization. These pathological changes, at the level of the spinal cord or even the central nerve at a higher level, can cause long-term changes in pain processing, leading to pain hypersensitivity inside and outside the injury area, further triggering central sensitization and chronic pain [16]. Clinically, neuropathic pain can be diagnosed by the ID pain scale, DN4 scale, LANSS scale, bedside assessment, and sensory signs assessment [17]. Studies have shown that, compared with the nonneuropathic pain group, the neuropathic pain group has significantly higher smoking rates, larger cuff tears, and much more severe rotator cuff muscle fatty degeneration [18].

The reason of rotator cuff injury limiting the movement of the shoulder joint was that it can lead to the shoulder kinematic change due to avoiding pain [19], in addition to structural damage directly interfering with kinematics. Patients with rotator cuff injury pain can still maintain the activities of the supraspinatus and infraspinatus muscles, but cannot activate all the deep muscles. They relied on the muscles around the scapula when doing forward flexion movements, and their strengths will decrease during external rotation [20]. During the healing process of rotator cuff injury, fibroblasts proliferate in large quantities and form granulation tissues with capillaries and deep inflammatory cells. The granulation tissue eventually becomes scar tissues. When the scars nearby the joints shrink, it may cause movement disorders. The lack of exercise further leads to atrophy of muscles, tendons, and other structures, which further leads to joint contracture and adhesion, and thus aggravate the limitation of shoulder mobility. The existence and disappearance of pain in the joints, the involved individual tendon, or muscle group will determine the progress and intensity of training [21]. Studies have shown that pain reduction can lead to significant improvements in shoulder muscle peak torque and power [22]. In our study, TENS treatment can improve the active mobility within the painless range.

One of the main hypotheses for the analgesic mechanism of TENS is based on the gate-control theory of pain. Electrical stimulation of large-diameter peripheral fibers inhibits the transmission of pain pulses in the spinal dorsal horn and, therefore, prevents pain signals from being transmitted into the brain. Other theories point out that electrical stimulation activates the descending inhibitory pathway from rostral ventromedial medulla (RVM) to spinal cord and mediates various neurotransmitters [23]. Among them, the low-frequency TENS (<10 Hz) can activate 5-HT, acetylcholine, and *µ*-opioid receptors in the spinal cord, while the high-frequency TENS (>50 Hz) can produce analgesic effects by activating acetylcholine and *δ*-opioid receptors in the spinal cord [24]. However, TENS may also cause adaptation, which can be prevented by adjusting the frequency modulation mode and increasing TENS intensity [25], for example, simulating acupuncture [26]. In addition, TENS has been proved effective in promoting the recovery of nerve function [27], which, therefore, may play a role in reducing neuropathic pains including the pain in shoulder joint.

As the analgesic effect of acupuncture is widely recognized [28], the way of placing electrodes on the wrist in this study was based on WAA theory [29] of TCM. According to WAA, the human body was divided into upper and lower halves, six longitudinal areas, corresponding to the six areas on each wrist and ankle, and by subcutaneously puncturing these areas, the pain can be effectively controlled and the tension can be relieved. In TENS, electrodes are usually placed on the corresponding painful dermatome to activate the large-diameter sensory fibers of the neuron and to enhance the inhibitory effect of SG cells on T cells to exert the analgesic effect. The common painful segment of rotator cuff injury is C6 [30], corresponding to the upper area 5 on the wrist. As pain suppression can also transmit information across segments, this may also be one of the neurobiological foundations of meridian signal transmission.

Compared with the control group, the VAS scores after the 1^st^ and 5^th^ treatment in the test group was significantly improved, indicating TENS on the wrist is effective in reducing pain due to rotator cuff injury.

Regarding the improvement of the shoulder joint mobility, there was no significant difference in the ROM and CMs between the two groups. This may be explained by the limited treatment period of our study; according to the literature, the abduction mobility of the rotator cuff that is difficult to repair has increased by 34.4° after 5 months of exercise training [31], the ROM is only slightly or not significantly improved in other directions, and it also takes months for proprioception and strength to recover.

In this study, we did not set up a sham TENS group because setting TENS at other frequencies or place electrodes at other locations may interfere with the results to a certain extent, and simply wearing the band without stimulation may not achieve the blind effect to the patients. We, therefore, used a randomized single-blind trial. In this study, the subjects did not know which group they were assigned to, and they were all told to treat with physical factors. The subjects of the two groups were assigned to different rooms and treated at different times. They had no contact with each other, which eliminate the placebo effect. This trial confirmed the analgesic effect of TENS on rotator cuff injury, but the difference and mechanism of pain relief between noxious pain and neuropathic pain remain unclear. Larger sample size and further basic and clinical research will be carried out in the future to explore the effect of electrical stimulation on muscles and nerves injury.

This experiment innovatively used the WAA theory of TCM combined with TENS to develop an analgesic band, which is effective and safe, and provides the reference value for the application of this device in other sports injury diseases. This combined treatment also provides ideas for the research and development of integrating Chinese and Western medicine rehabilitation equipment. It also provides support for portable and wearable physical therapy equipment in postrehabilitation treatment, especially individualized training at home.

## Figures and Tables

**Table 1:** General information of the two groups.

	Test group (*n* = 30)	Control group (*n* = 30)	Mean ± SD, *n* (%)	
			Value	*P*
Age	49.77 ± 10.96	53.33 ± 12.98	−1.15	0.255

Sex				
Male	12 (40%)	16 (53.33%)	1.07	0.301
Female	18 (60%)	14 (46.67%)		

Smoke				
Yes	3 (10%)	3 (10%)	0.00	1.000
No	27 (90%)	27 (90%)		

Shoulder surgery				
Yes	4 (%)	6 (20%)	0.48	0.488
No	26 (%)	24 (80%)		

Dominant hand				
Left	4 (13.33%)	1 (3.33%)	0.87	0.350
Right	26 (86.67%)	29 (96.67%)		

Injury location				
Left	17 (56.67%)	15 (50%)	0.27	0.605
Right	13 (43.33%)	15 (50%)		

MRI grade				
0	1 (3.33%)	0 (0%)	3.91	0.200
1	23 (76.67%)	18 (60%)		
2	6 (20%)	11 (36.67%)		
3	0 (0%)	1 (3.33%)		

Nature of onset				
Occult	21 (70%)	20 (66.67%)	0.08	0.781
Traumatic	9 (30%)	10 (33.33%)		

Duration				
0–3 months	11 (36.67%)	9 (30%)	3.12	0.595
3–6 months	10 (33.33%)	7 (23.33%)		
6–9 months	7 (23.33%)	8 (26.67%)		
9–12 months	0 (0%)	2 (6.67%)		
≥12 months	2 (6.67%)	4 (13.33%)		

**Table 2:** Comparison of VAS scores between the two groups.

	Test group	Control group	Median (*Q*_1_, *Q*_3_)	
			*Z*	*P*
VAS				
Baseline	5.00 (3.00, 6.00)	5.00 (4.00, 7.00)	1.01	0.315
After the 1^st^ treatment	4.00 (2.00, 5.00)^*∗∗*^	5.00 (3.00, 6.00)^*∗*^	2.81	0.000
After the 5^th^ treatment	3.00 (1.00, 4.00)^*∗∗*^	3.50 (2.00, 6.00)^*∗∗*^	2.00	0.045

Compared with baseline, ^*∗*^*p* < 0.05 and ^*∗∗*^*p* < 0.01.

**Table 3:** Comparison of shoulder mobility (ROM) between the two groups.

	Test group	Control group	Median (*Q*_1_, *Q*_3_)	
			Z	*P*
Flexion				
Baseline	99.50 (84.00, 118.00)	106.00 (90.00, 137.00)	0.90	0.366
After the 1^st^ treatment	107.50 (90.00, 120.00)^*∗∗*^	110.50 (92.00, 140.00)^*∗∗*^	0.99	0.320
After the 5^th^ treatment	120.00 (95.00, 135.00)^*∗∗*^	123.00 (100.00, 146.00)^*∗∗*^	1.67	0.095

Extension				
Baseline	35.00 (30.00, 43.00)	39.00 (30.00, 45.00)	1.16	0.247
After the 1^st^ treatment	40.00 (32.00, 45.00)^*∗*^	44.00 (36.00, 45.00)^*∗∗*^	0.57	0.570
After the 5^th^ treatment	45.00 (40.00, 48.00)^*∗∗*^	45.50 (40.00, 50.00)^*∗∗*^	0.25	0.799

Abduction				
Baseline	80.00 (70.00, 90.00)	78.50 (70.00, 90.00)	0.24	0.812
After the 1^st^ treatment	80.00 (75.00, 100.00)^*∗∗*^	83.00 (77.00, 95.00)^*∗∗*^	0.16	0.870
After the 5^th^ treatment	99.00 (88.00, 118.00)^*∗∗*^	95.50 (88.00, 118.00)^*∗∗*^	1.06	0.289

Adduction				
Baseline	30.00 (26.00, 40.00)	32.00 (20.00, 41.00)	0.33	0.743
After the 1^st^ treatment	33.00 (30.00, 40.00)^*∗∗*^	35.50 (25.00, 43.00)^*∗∗*^	0.09	0.927
After the 5^th^ treatment	39.00 (30.00, 44.00)^*∗∗*^	40.00 (32.00, 45.00)^*∗∗*^	0.03	0.976

Internal rotation				
Baseline	59.00 (38.00, 70.00)	59.50 (45.00, 70.00)	0.44	0.656
After the 1^st^ treatment	60.00 (45.00, 75.00)^*∗∗*^	61.00 (45.00, 71.00)^*∗∗*^	0.50	0.567
After the 5^th^ treatment	69.00 (50.00, 80.00)^*∗∗*^	67.50 (55.00, 78.00)^*∗∗*^	0.46	0.645

External rotation				
Baseline	25.50 (15.00, 40.00)	28.00 (18.00, 45.00)	1.16	0.244
After the 1^st^ treatment	36.50 (20.00, 45.00)^*∗∗*^	36.00 (20.00, 50.00)^*∗∗*^	1.30	0.193
After the 5^th^ treatment	42.00 (28.00, 50.00)^*∗∗*^	42.50 (32.00, 50.00)^*∗∗*^	0.94	0.346

Compared with baseline, ^*∗*^*p* < 0.05 and ^*∗∗*^*p* < 0.01.

**Table 4:** Comparison of CMs between two groups.

	Test group	Control group	Median (*Q*_1_, *Q*_3_)	
			*Z*	*P*
Pain				
Baseline	5.00 (5.00, 10.00)	5.00 (0.00, 5.00)	1.66	0.10
After the 5^th^ treatment	10.00 (5.00, 10.00)^*∗∗*^	7.50 (5.00, 10.00)^*∗∗*^	0.12	0.91

ADL				
Baseline	10.00 (10.00, 12.00)	10.00 (6.00, 12.00)	1.50	0.13
After the 5^th^ treatment	12.00 (12.00, 14.00)^*∗∗*^	12.00 (10.00, 12.00)^*∗∗*^	0.00	1.00

ROM				
Baseline	20.00 (14.00, 24.00)	22.00 (14.00, 24.00)	0.50	0.69
After the 5^th^ treatment	26.00 (18.00, 28.00)^*∗∗*^	26.00 (20.00, 28.00)^*∗∗*^	0.97	0.33

Strength				
Baseline	20.00 (15.00, 20.00)	20.00 (15.00, 20.00)	0.48	0.63
After the 5^th^ treatment	20.00 (20.00, 20.00)^*∗∗*^	20.00 (15.00, 20.00)^*∗∗*^	0.00	1.00

The total score				
Baseline	52.00 (45.00, 64.00)	50.00 (47.00, 59.00)	0.61	0.54
After the 5^th^ treatment	64.00 (56.00, 72.00)^∗∗^	62.50 (55.00, 70.00)^∗∗^	0.25	0.80

Compared with baseline, ^*∗*^*p* < 0.05 and ^*∗∗*^*p* < 0.01.

## Data Availability

The data used to support the findings of this study are available from the corresponding author upon request.
